# Optimization of Small-Scale Hydrogen Production with Membrane Reactors

**DOI:** 10.3390/membranes13030331

**Published:** 2023-03-14

**Authors:** Michele Ongis, Gioele Di Marcoberardino, Mattia Baiguini, Fausto Gallucci, Marco Binotti

**Affiliations:** 1 Dipartimento di Energia, Politecnico di Milano, Via Lambruschini 4a, 20156 Milan, Italy; 2 Sustainable Process Engineering, Department of Chemical Engineering and Chemistry, Eindhoven University of Technology, De Rondom 70, 5612 AP Eindhoven, The Netherlands; 3 DIMI—Dipartimento di Ingegneria Meccanica e Industriale, Università degli Studi di Brescia, Via Branze 38, 25123 Brescia, Italy; 4 Scuola Universitaria Superiore IUSS Pavia, Palazzo del Broletto, Piazza Vittoria 15, 27100 Pavia, Italy

**Keywords:** green hydrogen production, biogas, membrane reactors, fluidized bed, modelling

## Abstract

In the pathway towards decarbonization, hydrogen can provide valid support in different sectors, such as transportation, iron and steel industries, and domestic heating, concurrently reducing air pollution. Thanks to its versatility, hydrogen can be produced in different ways, among which steam reforming of natural gas is still the most commonly used method. Today, less than 0.7% of global hydrogen production can be considered low-carbon-emission. Among the various solutions under investigation for low-carbon hydrogen production, membrane reactor technology has the potential, especially at a small scale, to efficiently convert biogas into green hydrogen, leading to a substantial process intensification. Fluidized bed membrane reactors for autothermal reforming of biogas have reached industrial maturity. Reliable modelling support is thus necessary to develop their full potential. In this work, a mathematical model of the reactor is used to provide guidelines for their design and operations in off-design conditions. The analysis shows the influence of temperature, pressures, catalyst and steam amounts, and inlet temperature. Moreover, the influence of different membrane lengths, numbers, and pitches is investigated. From the results, guidelines are provided to properly design the geometry to obtain a set recovery factor value and hydrogen production. For a given reactor geometry and fluidization velocity, operating the reactor at 12 bar and the permeate-side pressure of 0.1 bar while increasing reactor temperature from 450 to 500 °C leads to an increase of 33% in hydrogen production and about 40% in HRF. At a reactor temperature of 500 °C, going from 8 to 20 bar inside the reactor doubled hydrogen production with a loss in recovery factor of about 16%. With the reactor at 12 bar, a vacuum pressure of 0.5 bar reduces hydrogen production by 43% and HRF by 45%. With the given catalyst, it is sufficient to have only 20% of solids filled into the reactor being catalytic particles. With the fixed operating conditions, it is worth mentioning that by adding membranes and maintaining the same spacing, it is possible to increase hydrogen production proportionally to the membrane area, maintaining the same HRF.

## 1. Introduction

A Membrane Reactor (MR) is an excellent example of process intensification. In the chemical industry, its application allows the replacement of a chain of downstream processes for product purification through the integration of membrane separation directly in the vessel where chemical reactions occur. Such integration is not always beneficial compared to the conventional reactor followed by the separation section since in the latter case the two steps can be optimized separately. MRs improve the system performance, especially in processes involving thermodynamically limited reactions, where the achievable conversion, at the same temperature, is higher than the equilibrium value of the conventional technology [1]. This happens in accordance with Le Chatelier’s principle: the system reacts to the change in concentration of products (due to the selective removal of one of them, in general), by increasing reactant conversion and then restoring a certain amount of products.

This apparently simple concept has the potential to be applied to several processes and to become a breakthrough in the process industry. In the European Union (EU)-funded project MACBETH, MR technology is applied to four different processes in different areas: hydrogen production in the energy sector, propane dehydrogenation and hydroformylation in the chemical industry, and ω-3 fatty acids enrichment in biotechnology [2]. Among them, the most studied process where MRs are close to becoming industrial practice is hydrogen production through methane/hydrocarbon reforming. Potential methane sources are natural gas or biogas (BG)/biomethane, while possible plant configurations are both the conventional steam reforming process—where the heat duty of the endothermic reactions is provided by tubes immersed in the reactor where hot flue gases flow (not directly in contact with the reactive mixture)—and autothermal reforming (ATR)—where the heat is provided by the combustion of part of methane fed into the reactor, via the addition of air/oxygen in the reactive mixture. Both packed bed and fluidized bed reactor configurations have been investigated in the literature [3]. A fluidized bed should allow a reduction in bed-to-membrane mass transfer limitation (known as concentration polarization (CP) losses), a reduction in the pressure drops, and, also importantly, working at approximately uniform temperature conditions, thanks to solid motion that allows rapid distribution of the heat [4]. As will be pointed out in this article, reactor temperature has a strong influence on hydrogen production, and its uncontrolled increase could lead to serious damages to the membranes, reducing their selectivity and operating life: for this reason, the fluidized bed configuration is currently the most considered for industrialization of MRs for methane reforming. In MACBETH, the two prototypes of MRs applied to hydrogen production are both fluidized bed catalytic membrane reactors (FBMRs), but they use a different feedstock, and they have different configurations and sizes: one is for biogas autothermal reforming, aiming for the production of about 100 kg_H2_/day, while the other one is for natural gas steam reforming, with a slightly smaller size of around 40 kg_H2_/day. These reactors come after several years of studies on membranes, fluidizable catalysts, and their optimal integration in a single vessel, testified by several articles and EU-funded projects [5,6]. The ambitious aim of the MACBETH project points out how MR technology is close to demonstrating its industrial potential for small-scale hydrogen production. Moreover, the concept has the potential to be scaled up using bigger fluidized beds or modular configurations.

A digression on the importance of hydrogen production is unnecessary in the current energy context since many reports published yearly assess its impact and underline the importance of a growing share of hydrogen in many different sectors [7,8]. The same discussion holds for the importance of increasing the share of low-carbon hydrogen [9] since production from fossil fuels (mainly natural gas and coal) without carbon capture, use, and storage (CCUS) is today the most commonly used method for dedicated hydrogen production (non-dedicated production, which accounts for about one-third of total hydrogen production, comes from processes where H_2_ is a by-product in a process designed primarily for other products). Overall, less than 0.7% of current hydrogen production comes from renewable sources or fossil fuel-based plants with CCUS. Demand for pure hydrogen is about 70 million tons, mainly in the oil industry and ammonia production for fertilizers, and about 4 Mt is required for chemicals, metals, electronic and glass industries, and transportation. Oil demand is expected to increase by 2030 in all its current applications, which are mainly industrial, but opportunities exist in other sectors such as transportation (road, aviation, rail, and maritime), as a fuel for heat supply in buildings, and for power generation [8]. It is clear that these perspectives make room for emerging technologies to increase the share of low-carbon hydrogen production.

In conclusion, MRs can be considered a full-fledged promising technology in the hydrogen market interests for the near future. In this context, modelling activity is a fundamental support for MR design and for understanding their behavior in off-design conditions. This especially holds for FBMR, where the description of the catalytic reactions and of the membrane separation goes together with the behavior of the fluidized bed, and then process parameters are related to each other in a complex way. In the reactor model, a fluid dynamic description should be included to take into account as many effects as possible. The literature presents several works on FBMR modelling for hydrogen separation, at the building block scale, at the reactor scale, and at the process scale (considering the overall system which includes the MR). In the first category are included the works on catalysts [10] and membranes [11] (developed to work in the fluidized bed) and the research on fluid dynamic behavior, such as bubble properties [12] and concentration polarization losses [13,14]. These works were mainly based on experiments, with the aim to have detailed and accurate models that could be also further included in an overall FBMR model. At the reactor level, some modelling works were performed to reproduce the experimental results with lab-scale prototypes [15,16,17] and to study the trends of some parameters throughout the reactor [18,19]. At the system level, works in the literature are mainly modelling predictions on the potential performance of the systems, to compare different feedstocks [20,21] or to optimize the system performance [21,22,23]. The present article arises in this context since many materials are available on FBMR modelling but it is still difficult to synthesize this knowledge about the impact of different parameters on reactor design and operation.

In more detail, in this work, a mathematical model of a fluidized bed membrane reactor, developed in Aspen Custom Modeler (ACM) in previous works [18,20], is improved and used to investigate the performance of the reactor in different operating conditions, to provide guidelines for the design stage, and to predict reactor behavior in off-design conditions due to variations in operating variables, such as temperature and pressure, or in the fluidization velocity of the reactor. The reactor is fed by biogas, and the sizes studied are similar to the real prototype under construction in MACBETH. Section 2 presents the detailed methodology of the work. This analysis of the influence of operating conditions is presented in Section 3, together with geometric considerations on the reactor. Guidelines for reactor design are provided in Section 4.

## 2. Methodology

### 2.1. FBMR Model and KPIs

The FBMR model used in this work comes from the evolution of previous FBMR models [18], and a detailed description of its latest version can be found in a work from the same authors [21]. In the following, only the main features of the model are thus reported. It is a 1D continuous model where an overall energy balance and the material balances for all species involved are solved. The model assumes the presence of 2 phases: emulsion and bubble phase. Based on the original two-phase model, the emulsion phase is always in minimum fluidization conditions (solid particles suspended since the drag force of the inlet gas and floating are perfectly balanced by gravity), and all the gas in excess passes through the bed in bubble form. Bubbles are described as spheres without catalytic particles in them, except for the bottom phase of the bubble, called the wake, which has solid particles in it. In this analysis, the wake fraction is assumed to be in minimum fluidization conditions, so it can be considered part of the emulsion, even if its value depends on the bubble size at a certain height of the bed. In both emulsion and bubble phases, material balances are used to calculate the variation in moles of all the components due to chemical reactions (only in the emulsion) and membrane permeation (from both phases), as well as transfer coefficients between the two phases due to difference in component concentrations. An overall energy balance verifies the autothermal behavior of the bed. Kinetic and permeation models are reported in [21]. The involved chemical reactions are the following:

R.1 $$\begin{array}{cc} CH_{4}+H_{2}O\;↔CO+3H_{2}\; & \Delta H_{@298\;K}^{0}=206.1\;\mathrm{kJ/mol} \end{array}$$

R.2 $$\begin{array}{cc} CO+H_{2}O\;↔CO_{2}+H_{2}\; & \Delta H_{@298\;K}^{0}=-41.15\;\mathrm{kJ/mol} \end{array}$$

R.3 $$\begin{array}{cc} CH_{4}+2O_{2}\to CO_{2}+2H_{2}O\; & \Delta H_{@298\;K}^{0}=-802.3\;\mathrm{kJ/mol} \end{array}$$

The equations used to characterize the model are reported in Appendix A. They include the kinetic part, with reaction rate calculations, the permeation equations, and the description of the fluidization behavior. Material balances are performed for each chemical species involved in both emulsion and bubble phases.

The behavior of the dense region of the reactor depends on many operating parameters interconnected with one another. Pressures and flow rates are regulated externally through valves and compressors, while system temperature is maintained at a desired set value by feeding the correct amount of air, which provides oxygen for methane combustion. Temperature and pressure influence the specific volume of the gas inside the reactor, thus influencing its velocity and consequently the fluidization regime. Higher velocities increase the bubble fraction and then the gas bypass, reducing methane conversion. These are only examples to show how system variables are interrelated and why it is important to study their effect on reactor behavior. The number of variables to be considered increases if reactor design is performed, and thus the reactor geometry and the number of membranes is not yet defined.

To assess the performance of the FBMR in different operating conditions, a key performance indicator (KPI) has to be defined. In this analysis, the efficiency parameter at the reactor level is called *hydrogen recovery factor* (HRF) and is defined as the ratio of pure hydrogen separated over the maximum theoretical amount of hydrogen that can be separated if (i) all methane fed (except the share burned for autothermal behavior) is converted according to the previous reactions, so 4 moles of hydrogen is produced per mole of methane, and (ii) all hydrogen produced is separated from the membrane. This second condition is ideal even in case of a complete chemical reaction since it would require zero pressure in the vacuum side.

(1) $$HRF=\frac{{\dot{n}}_{H_{2,perm}}}{4\cdot \left({\dot{n}}_{CH_{4,in}}-{\dot{n}}_{CH_{4,ox}}\right)}$$

Another performance indicator is the pure *hydrogen production*, expressed in absolute terms of kg of hydrogen separated through the selective membranes per unit time (thus, hydrogen produced in the reactions that ends up in the retentate flow is not accounted for in this indicator). Each working condition of the reactor is represented by a point in the HRF–*hydrogen production* plane, hereafter called the *performance chart*. Performance charts are illustrated in detail in Section 3.1.

### 2.2. Model Validation

**Figure 1:**
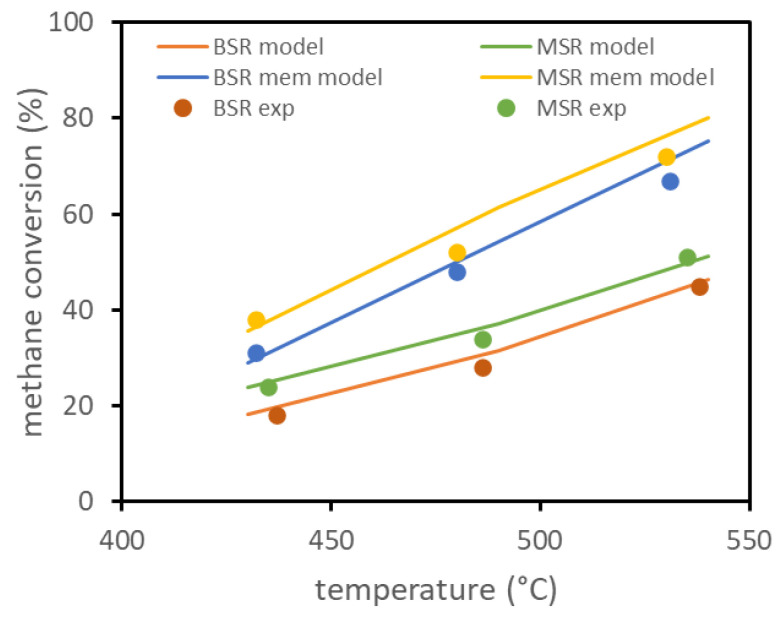
FBMR model validation based on lab-scale experimental data reported in literature.

The model has been preliminarily validated on experimental results available in the literature for biogas ATR in a fluidized bed membrane reactor. The only results available, reported in [13], refer to a lab-scale reactor with a diameter of about 4.3 cm, with one immersed membrane of about 14 cm length and 1.4 cm diameter. The same reference reports the experimental setup, together with parameters for the membrane permeation model and for the kinetic model. The experiments evaluated the conversion of methane at different reactor temperatures for two different compositions, with and without the presence of the membrane inside the reactor. The experiments were performed with a total feed of 3.6 NL/min, firstly considering a molar composition of 10% $CH_{4}$, 7% $CO_{2}$, 30% $H_{2}O$, and 53% $N_{2}\;$(BSR case) and then considering a composition of 10% $CH_{4}$, 30% $H_{2}O$, and 60% $N_{2}$ (MSR case). Experimental results, with and without the presence of the membrane, and the results obtained using the model are reported in Figure 1 and show the good fitting of the model. Experiments were performed with the reactor at 3 bar and permeate side at 0.1 bar.

### 2.3. Assumptions

The reactor feed is a mixture of BG, pure steam, and air. The BG flow rate is a variable that is freely changed, while the steam flow rate changes accordingly in order to set a fixed steam–carbon ratio (SCR, the ratio between the molar flow of steam and the molar flow of methane) at the beginning of the membrane region (5 cm from the distributor plate of the reactor). In addition, the air flow rate changes in accordance with biogas and steam flow rates in order to maintain the autothermal behavior of the reactor. BG and air composition in terms of molar fractions assumed in this analysis are reported in Table 1.

BG composition is representative of a typical anaerobic digestion plant. Biogas is fed into the membrane reactor in saturated condition (at 25 °C, 1 bar), with a methane content of approximately 58.1%, resulting in an LHV of 17.8 MJ/kg. Air is assumed to be a binary mixture of nitrogen and oxygen.

Compared to previous works, the parameters for the membranes’ characterization have been updated. In this work, the so-called *double-skin membranes* are considered [24]: these membranes are different from the previous ones (Pd/Ag selective layer over cylindrical ceramic support) due to the presence of an additional mesoporous ceramic protective layer to improve the membranes’ resistance to collisions with catalytic particles. These ceramic-supported dense metal membranes are preferred to other alternatives, such as dense ceramic membranes, thanks to wider investigations for applications in fluidized beds and higher hydrogen fluxes.

Membrane parameters are reported in Table 2 and taken from [11]. These parameters are used to calculate hydrogen flux through the membranes (see Equation (A6) in Appendix A). Due to the very high selectivity of dense membranes, in the model, it is assumed that only hydrogen crosses the membranes (so an ideal infinite selectivity), and then the permeate side is pure hydrogen. The effect of membrane ceramic support is neglected, but its influence can be investigated in the analysis of permeate pressure increase. Due to typical reactor temperatures higher than the critical temperatures of most of the mixture components, the gas mixture within the reactor is assumed to be an ideal mixture of ideal gases.

In addition, the catalyst formulation has been updated. In this work, the catalyst considered is a rhodium-based catalyst, with a formulation of 1.6 weight percentage of rhodium over a zirconia oxide support (ZrO_2_). The formulation is taken from [10], as are the parameters (Table 3) and the relative reaction rate expressions (Equations (A2) and (A3) in Appendix A).

**Figure 2:**
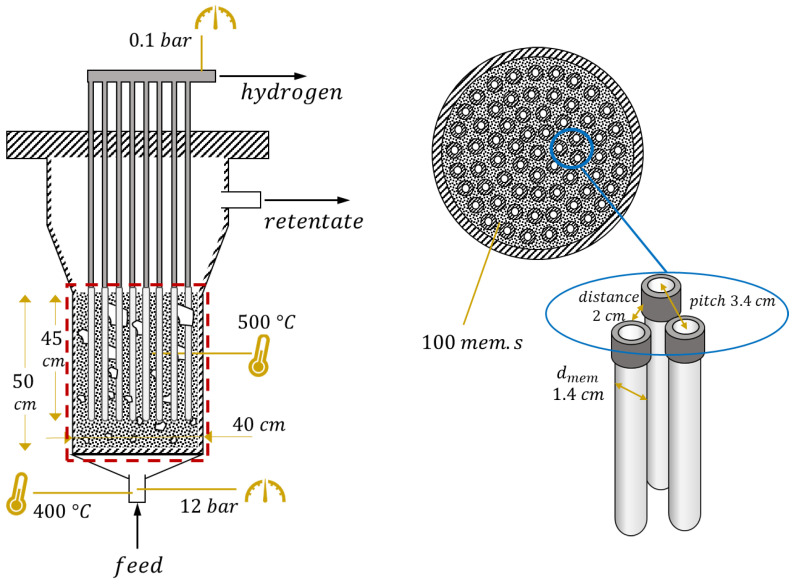
FBMR layout with the values of geometric and operating parameters at nominal conditions. Dense region modeled is in the red square, where the catalyst (black dots) and membranes (in white) are present.

The reactor layout and values of parameters in nominal conditions are shown in Figure 2. Membrane geometry and operating conditions represent the current state of the art of the technology. Feed inlet temperature is set to 400 °C, a reasonable value considered not too low, thus avoiding strong thermal gradients within the reactor, and not too high, since the feed mixture is in general preheated from retentate and permeate flow and there is the risk of preignition of biogas–air mixture if temperatures are too high. Temperature is assumed constant in the reactor due to the mixing which occurs in the fluidized bed. The value of 500 °C is a compromise between reactive performance, which requires high temperature, and membrane stability, which can be seriously affected above 525 °C. The reactor pressure value (12 bar) comes from preliminary optimizations [23], as does the permeate-side vacuum pressure (0.1 bar). Their difference is strictly related to the driving force for hydrogen permeation through the membrane. SCR is calculated at the beginning of the membrane region (after the oxidation and some reforming), not at the reactor inlet, and the set value is 3, in order to ensure a negligible carbon deposition on the catalyst [10]. Regarding the catalyst, the total amount of solids is directly computed by the model equations to guarantee a correct fluidization regime. However, it is possible to operate on the ratio between catalytic particles (meaning support with active metal on the top) and filler particles (made of support material only). In a nominal situation, it is assumed that all the solids are made of catalytic particles. Membrane length, 45 cm, and external diameter, 1.4 cm, are values taken from the membrane developer [24]. The internal diameter of the inner tube where hydrogen is collected is 0.7 cm. The value of membrane distance (side-to-side) has been set to 2 cm, thus leading to a membrane pitch of 3.4 cm, based on experimental results which showed that below this value the hydrogen concentration at the membrane surface can be strongly reduced by the permeation through neighbor membranes [25]. In these conditions, it is also assumed that CP losses can be neglected, even if a potential range for their impact is provided in a following analysis. All these parameters will be investigated in the reactor-level analysis, except for membrane diameter which has always been kept fixed. In all the analyses, the parameters are changed one at a time, while all the others maintain their nominal values reported in Figure 2.

The analysis at the reactor level includes both operative and geometric considerations, leading to design guidelines. The first analysis, presented in Section 3.2, considers the reactor geometry (including membrane geometry and number) fixed and provides guidelines about off-design operations of the reactor, showing the influence of reactor temperature, pressures, SCR, CP losses, and catalyst amount on reactor performance. Section 3.3 presents a study of geometric considerations about membrane number, spacing, and length. Based on all these considerations, some guidelines for FBMR design are presented in Section 4. Geometric considerations include the influence of membrane length and number and, consequently, the length-over-diameter ratio of the reactor.

## 3. Reactor Analysis

### 3.1. Performance Charts

**Figure 3:**
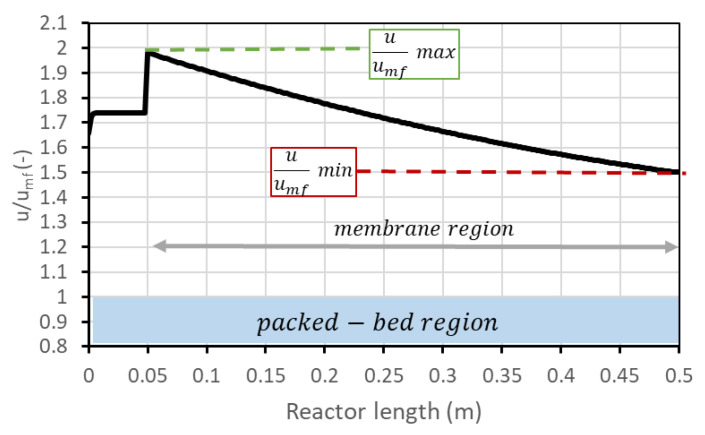
Trend of $u/u_{mf}$ along the reactor. After 5 cm from the bottom, the membrane region starts.

As mentioned, the performance of the FBMR is expressed in terms of HRF and hydrogen production. For a defined set of parameters (reactor temperature and pressure, permeate-side pressure, SCR, amount of catalyst, number of membranes, reactor geometry), it is still possible to operate at different points in the HRF–*hydrogen production* chart by changing the BG flow rate and steam and air flow accordingly (steam to maintain SCR; air to maintain ATR conditions). The different points then correspond to different fluidization velocities. What happens in general is that hydrogen production increases with BG flow rate, but HRF decreases. It is then possible to decide to operate the reactor in a region with higher production but also with higher unconverted methane content in the retentate, which is a loss but can also be necessary if additional heat is required by the plant. BG flow rate can be varied in accordance with FBMR boundaries: high values mean a high volumetric flow rate which results in a higher velocity of the inlet gas. At higher velocities, the bubble fraction increases, and gas drag force can become enough to bring catalyst particles outside the reactor, which is of course an undesired effect. In this analysis, the maximum BG flow rate is set to be reached when the maximum value of $u/u_{mf}$ within the reactor is 5, to be conservative in regard to the risk of catalyst removal. Parameter $u/u_{mf}$ represents the ratio between the superficial velocity of the gas (volumetric flow rate over cross-section) and minimum fluidization velocity (which is gas velocity to have minimum fluidization conditions in the bed) and is an indicator of the fluidization regime. $u/u_{mf}$ is not constant inside the reactor, and in general has its maximum at the beginning of the membrane region (due to cross-section decrease) and its minimum at the outlet (due to hydrogen permeation which reduces volumetric flow). The trend of this variable along the reactor is shown in Figure 3.

**Figure 4:**
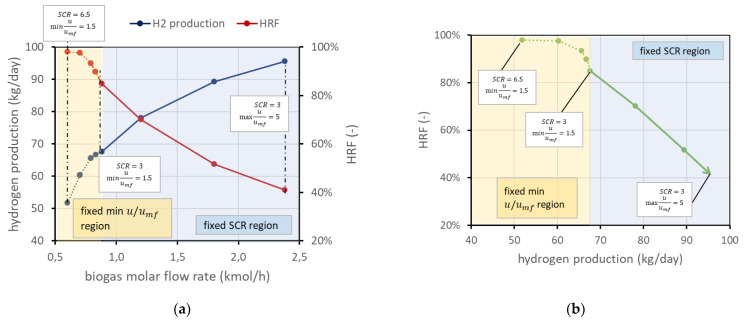
(**a**) Trends of HRF and hydrogen production for different BG flow rates in nominal conditions. (**b**) Performance chart corresponding to the same working points of (**a**). In both panels, the solid line represents the working points with fixed SCR and increasing velocity in the direction of the arrow. The dotted line is the region where velocity is fixed and then SCR has to increase to provide enough steam to fluidize the bed.

On the other side, a low BG flow rate leads to lower velocities. To guarantee a safety margin with respect to the risk of bed defluidization, the reactant flow rate is selected to have a minimum velocity 50% higher than the required minimum fluidization velocity (i.e., $u/u_{mf}$ = 1.5). This margin has been chosen since radial effects are not considered with a 1D model, and velocity next to the vessel wall is generally lower due to friction. Due to the variation in volumetric flow related to the chemical reactions and to the permeance through the membranes, minimum flow velocity typically occurs at the reactor outlet, but in case very low permeation occurs, it can also be found at the reactor inlet. It is still possible to use an even lower BG flow rate, but then, since the velocity should be fixed (minimum $u/u_{mf}$ in reactor 1.5), the steam flow (then the SCR) should increase accordingly. For clarity, these considerations are reported in Figure 4a. The same information can be reported in a different type of chart, eliminating the information on biogas molar flow rate, ending up with the performance chart in Figure 4b. These charts refer to the nominal condition as defined in Figure 2.

The performance chart is a tool that allows the working points to be clearly displayed and differences among cases with different parameters to be easily compared. Each case, defined by the set value of the abovementioned parameters (reactor temperature and pressure, permeate-side pressure, SCR, amount of catalyst, number of membranes, reactor geometry), is represented by a line in the performance chart. The arrow in the performance chart goes in the direction of increasing biogas flow rate (lines with the same flow rate in the chart are similar to the black dashed lines), and the arrowhead is the point where maximum $u/u_{mf}$ in the reactor is equal to 5.

The trend of the line in the performance chart can be explained based on the reactor physics: in the fixed SCR region, increasing the BG flow rate allows higher production to be reached because, obviously, more methane is available for the reaction. However, higher velocity means a lower residence time, and thus a minor methane conversion, which results in a lower HRF. In the dotted region with fixed velocity, points on the left have an excess of steam, which is a reactant in reforming reactions and then allows a higher methane conversion. However, there is of course a consumption associated with additional steam production that can make an undesired excess of SCR.

### 3.2. Influence of Operating Conditions

An understanding of the effect of the operating conditions on the reactor performance is necessary for the operation of the reactor, once the geometry and the number of membranes have already been set. In this case, it can be important to understand their effect to know the influence, for example, of thermal gradients within the reactor. Moreover, it helps with off-design calibration of the reactor, in case it is decided to operate at different conditions to increase or decrease hydrogen production and/or HRF.

**Figure 5:**
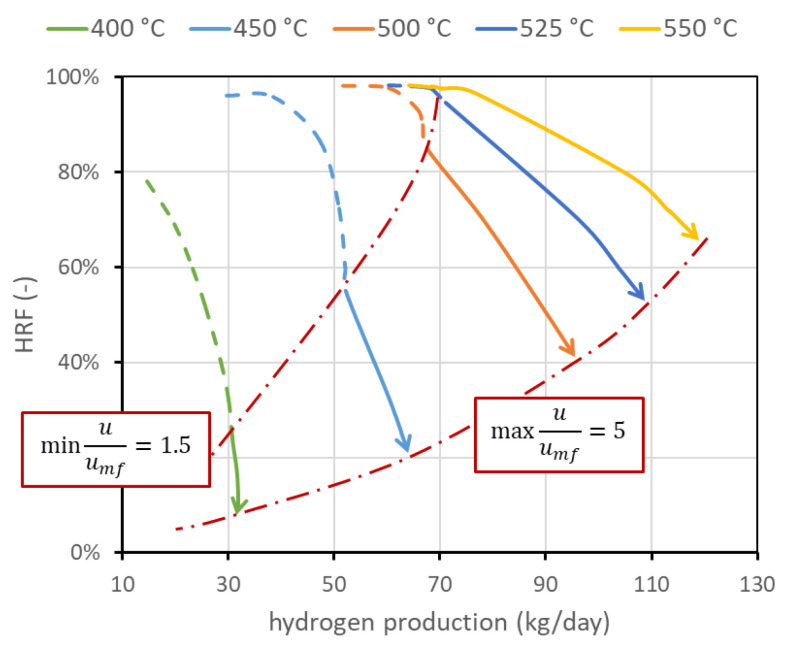
Performance chart with lines at different reactor temperatures. It appears clear that temperature strongly affects performance, and how it is important to increase its value to increase both HRF and hydrogen production. Temperature upper limit is due to membrane stability. Nominal value is 500 °C.

The first operating condition is the reactor temperature $T_{R}$. As stated, the temperature of the mixture (BG, air, and steam) is set to 400 °C. A further increase in the temperature is given by the heat provided by exothermic methane oxidation, which is measured out to balance the heat required by the endothermic reforming reactor and the heat necessary for the temperature increase. The temperature within the reactor can be assumed constant since the reactor is a fluidized bed, and rapid solid movement should be able to quickly distribute the heat [4]. The temperature gradient at the reactor inlet is also neglected since combustion reactions seem to be very fast compared to the other reactions and are completed in the first few centimeters. The nominal temperature has been set to 500 °C. This value is representative of the state of the art and is a trade-off between the reforming reaction kinetics, which needs high temperatures to have a reasonable conversion, and membrane stability, which is negatively affected by high temperature. Values investigated are in the range of 400–550 °C. The corresponding performance chart is reported in Figure 5. This figure also presents the lines with the same minimum velocity. In any case, if the system is regulated to work at the same fluidization regime or at a fixed biogas flow rate, an increase in temperature is beneficial for both HRF and the amount of hydrogen separated. In particular, from the kinetic point of view, it is well known that an increase in operating temperature improves reactant conversion and then hydrogen production. Additional hydrogen production, and then its higher molar fraction, increases the driving force and then increases hydrogen flux. Moreover, permeance through the membranes increases with temperature, leading to an additional improvement.

**Figure 6:**
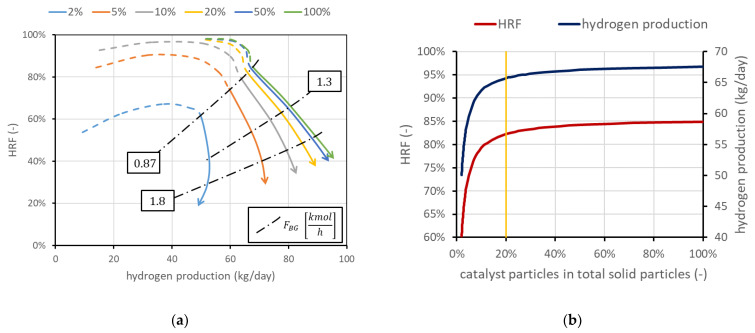
(**a**) Performance chart obtained for different percentages of catalytic particles over total solid particles in the reactor. Nominal value is 100% catalyst. (**b**) Impact of catalyst percentage in solid particles on HRF and hydrogen production for points at minimum $\frac{u}{u_{mf}}=1.5$.

The mass of solids inside the reactor is a variable whose value is directly provided by the model once the density and size are set. Fluid dynamics is in fact related to the particle size and density, and to obtain the required void fraction in the emulsion phase (determined by a correlation in the model), a precise mass of solids is required. However, not all the particles have to be catalytically active, meaning that a part of them can be only made of the support without the active metal on the surface. The amount of catalyst can be a constraint (due to high cost, for example) or can represent a situation in which part of the catalyst is deactivated due to carbon deposition. Moreover, it is interesting to determine the amount of catalyst needed to reach a certain production, avoiding an excess of catalytic material whose effect on performance is negligible. Results obtained using the FBMR model are shown in Figure 6a, in which are also reported three iso-biogas molar flow rate lines, for a deeper understanding of the performance charts. The different curves refer to different percentages of catalytic particles over total solid particles. In the cases investigated, the total mass of solids is in the order of magnitude of 50 kg. The results show that the amount of catalyst can influence the shape of the curve, and then the convenience of working with high SCR in the dashed-line region. Moreover, the chart clearly shows how the catalyst effect tends to saturate: the difference from 50 to 100%, even if the catalyst is doubled, is negligible. When this happens, it can be said that the catalyst amount is enough to reach a sort of equilibrium condition. Equilibrium is not in its strict meaning since in MRs, the presence of membranes continuously shifts the equilibrium and then additional conversion is always possible. A real equilibrium condition can be reached if the catalyst and membrane area are enough to produce and remove all the hydrogen until its partial pressure in the retentate side at chemical equilibrium is the same as the permeate-side pressure, with a complete methane conversion. Nominal conditions have been selected to have 100% catalyst, in order to avoid its influence in the investigated cases. However, it is in general convenient to work with a lower amount of catalyst due to its high cost. Considering for example the points at $u/u_{mf}$ = 1.5, which in this case are also the points connected by the line at 0.87 kmol/h of biogas, HRF and hydrogen production can be plotted as a function on the percentage of catalytic particles over the total solid particles. Results are shown in Figure 6b, which shows that in these conditions, a fraction of 20% leads to a reduction in hydrogen production and HRF of about 3%, while catalyst cost is reduced by 80%.

**Figure 7:**
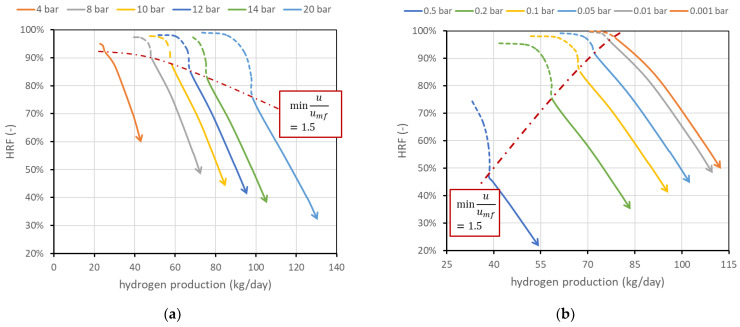
(**a**) Effect of reactor pressure on FBMR performance. (**b**) Effect of permeate-side pressure. Nominal values are 12 bar within the reactor and 0.1 bar in the permeate side.

The effect of reactor pressure $p_{R}$ on reactor performance is less straightforward since two competitive effects occur: the methane conversion in reforming reaction decreases (as the reaction occurs with an increase in the number of moles), while the driving force for permeation, related to the hydrogen partial pressure within the reactor ($p_{H_{2},reactor}$), increases as hydrogen flux $J_{H_{2}}$ through the membrane is regulated by Richardson’s equation (see Equation (A6) in Appendix A). Results are shown in Figure 7a. The chart shows how an increase in reactor pressure allows the production of more hydrogen (curves shifted towards the right) but, in a certain fluidization regime, with lower efficiency. More specifically, if the reactor is regulated to work at constant fluidization velocity, an increase in reactor pressure reduces the volumetric flow rate of the retentate, since specific volume decreases. Then, a higher feed flow rate is required to maintain the same velocity. Since driving force increases and BG fed increases, hydrogen production increases. However, the membrane-area–methane ratio decreases, and this leads to a reduction in HRF (a lower membrane area available for unit flow of methane). On the other hand, if the system is regulated to maintain a constant BG flow rate, since volumetric flow is reduced by an increase in reactor pressure, the velocity inside the reactor decreases. This leads to an increase in residence time and then an increase in methane conversion; then, both HRF and hydrogen production increase. This effect occurs only if the initial working point was already at high velocity; otherwise, it is necessary to work in the dotted region (then increase SCR).

Figure 7b shows the effect of permeate-side vacuum pressure $p_{perm}$. Since it is assumed that only pure hydrogen is present in the permeate side, its partial pressure, which appears in Richardson’s equation, is equal to the vacuum total pressure, and its reduction increases hydrogen flux. The effect leads in general to a saturation effect and then to a trade-off between the complexity of generating high-vacuum conditions and the benefits of such low permeate pressure. On the other hand, the influence of the support (which is neglected in this analysis) can be important depending on the support type. The performance chart shows how its influence should be studied, since the difference in reactor performance moving, for example, from 0.1 to 0.2 bar in the vacuum side is relevant: at the same minimum fluidization velocity, HRF can be reduced from 85 to 74% and hydrogen production can be reduced from 67.6 to 57.6 kg/day. The two effects are in this case related since vacuum-side pressure has no direct influence on the reforming reaction and volume flow rate within the reactor, and then the minimum fluidization velocity is obtained at the same BG flow rate. Then, when the permeate pressure increases, driving force and hydrogen production decrease. Less hydrogen removed for the same BG input means a lower recovery factor.

**Figure 8:**
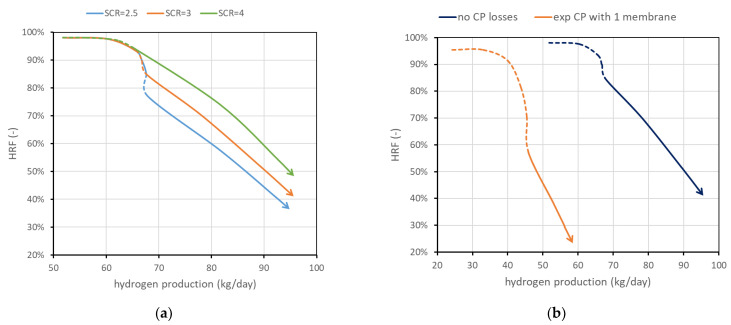
(**a**) Effect of SCR. (**b**) Effect of CP losses (ideal vs. severe case). Nominal value of SCR is 3 and CP losses are neglected.

Another parameter investigated is SCR. Working with more steam (higher SCR) leads in general to an improvement in the reactor performance since steam is one of the reactants and its presence allows working with a lower amount of BG (then increasing membrane area for unit methane) and then increasing HRF, maintaining an admissible fluidization velocity. Results are shown in Figure 8a. It is, however, evident that higher SCR means additional heat for steam generation, and the convenience should be carefully evaluated at the system level.

Figure 8b shows the possible influence of concentration polarization losses. CP losses have an important influence at the lab scale, and it is expected that their influence is strongly reduced at the scale investigated in this work, since values obtained at the lab scale typically are obtained with one to a few membranes immersed in a large bed of particles, and then gas bypass and large distances between the bulk and the membranes result in high CP values. However, to provide some boundary of their influence, two cases are reported in the performance chart: neglecting CP losses against performance considering a mass-transfer resistance obtained experimentally in [13] working with a lab-scale reactor with one membrane. Assuming this is a severe-reduction case, it can be stated that CP influence should fall between the two curves reported.

**Figure 9:**
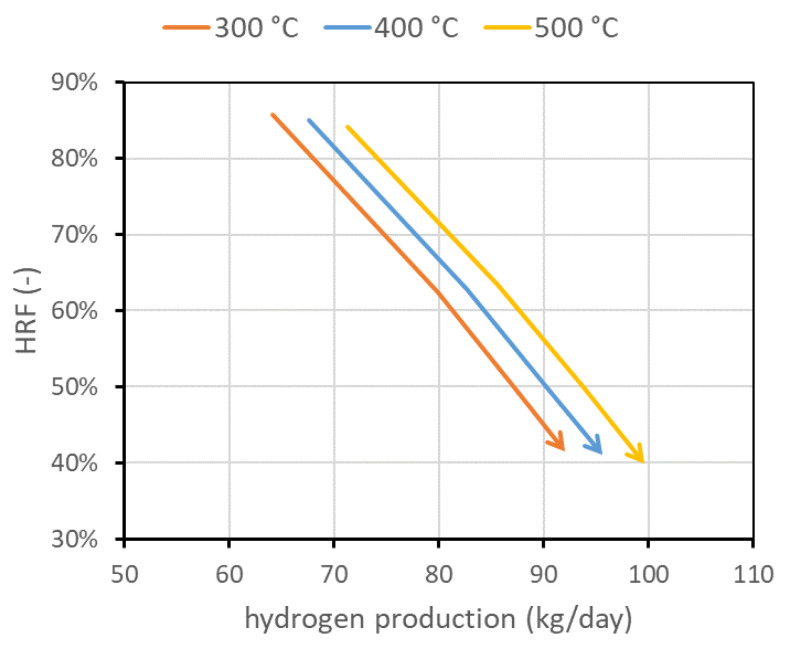
Performance chart obtained for different feed temperatures, maintaining reactor temperature at 500 °C in all cases.

The last investigated parameter is the temperature of the reactor feed mixture of biogas, steam, and air, called $T_{feed}$. Here, 400 °C represents the nominal value. For lower temperatures, additional oxygen consumption is required. This also means that more moles of steam are produced by combustion reaction, and then less steam should be fed to the reactor to obtain a fixed SCR. The trend is the opposite at higher temperatures. Globally, to maintain a desired fluidization regime, the case at higher temperatures requires slightly more biogas than the cases at lower temperatures. This is reflected in a slightly smaller HRF but additional hydrogen production, as shown in Figure 9. In the figure, only the region at fixed SCR (solid line) is shown, with the $u/u_{mf}$ always going from 1.5 to 5 (arrowhead).

### 3.3. Geometric Considerations

Beyond operational considerations, it is interesting to also consider the geometric effects and the variations in membrane area. All these considerations, together with the ones presented in the previous section, should be considered for the design of the reactor.

**Figure 10:**
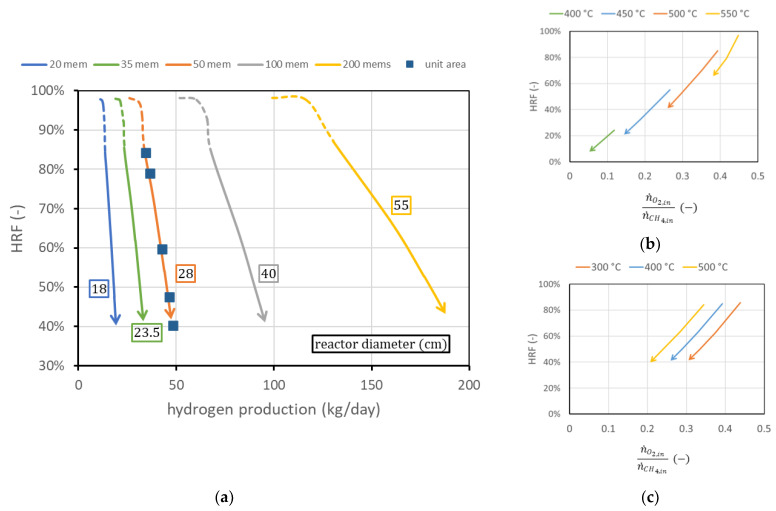
(**a**) Performance charts obtained for different numbers of membranes (with the same spacing) fitted into the reactors (of different diameters). Labels indicate the reactor diameter $d_{R}$. (**b**) Ratio between oxygen and methane to obtain autothermal behavior for different reactor temperatures at $T_{feed}$ = 400 °C and (**c**) for different $T_{feed}$ (bottom) at reactor temperature $T_{R}$ = 500 °C.

The first analysis considers the membrane length and pitch fixed, as well as considering all the operating conditions fixed, and shows the effect of the variation in the number of membranes. The reactor diameter will change accordingly to guarantee constant spacing between the membranes. Results are shown in Figure 10a. The effect of additional membrane area is basically a shift to the right of the curve in the performance chart. Each curve can also be plotted in terms of hydrogen production per unit of membrane area: in this case, all curves collapse on the 50-membrane curve in the figure (since 50 of the membranes used have a superficial area of about 1 m^2^). In other words, adding membranes while maintaining the same spacing is equivalent to having different reactors in parallel: HRF is the same once fluidization is fixed, and hydrogen production is proportional to the membrane area. This also implies that from the knowledge of the curve per unit membrane area, it is possible to predict the reactor performance for any different membrane area obtained by adding membranes at constant spacing. It is also possible to derive, from the chart, the amounts of biogas and air necessary to work in ATR conditions. The only additional information needed is the ratio between oxygen (then air) and methane (then biogas) to avoid any duty of heat from outside, as is shown in Equations (2) and (3).

(2) $$HRF=\frac{{\dot{n}}_{H_{2,separated}}}{4\cdot \left({\dot{n}}_{CH_{4,in}}-{\dot{n}}_{CH_{4,oxidated}}\right)}=\frac{{\dot{n}}_{H_{2,separated}}}{4\cdot \left({\dot{n}}_{CH_{4,in}}-\frac{{\dot{n}}_{O_{2,in}}}{2}\right)}=\frac{{\dot{n}}_{H_{2,separated}}}{4\cdot {\dot{n}}_{CH_{4,in}}\cdot \left(1-\frac{1}{2}\cdot \frac{{\dot{n}}_{O_{2,in}}}{{\dot{n}}_{CH_{4,in}}}\right)}$$

(3) $${\dot{n}}_{CH_{4,in}}=\frac{{\dot{n}}_{H_{2,separated}}}{HRF\cdot \left(1-\frac{1}{2}\cdot \frac{{\dot{n}}_{O_{2,in}}}{{\dot{n}}_{CH_{4,in}}}\right)}$$

Then, for a certain point in the performance chart (${\dot{n}}_{H_{2,separated}}$; HRF), the amount of methane to be fed is determined from the knowledge of the ratio $\frac{{\dot{n}}_{O_{2,in}}}{{\dot{n}}_{CH_{4,in}}}\;$. This value in general does not depend on reactor geometry, the number of membranes, or the permeate-side pressure. There exists a small dependence on reactor pressure and on SCR, which can be neglected in the first approximation. The only relevant parameters that influence the amount of oxygen required to maintain autothermal behavior are reactor temperature and feed inlet temperature. Their influence is shown in Figure 10b,c, for the region at fixed SCR (solid line). The steam flow rate required is influenced both by the biogas flow and by the oxygen–methane ratio. In general, more oxygen per unit methane is required and more steam is produced by combustion reaction, and then less steam has to be provided to the reactor. The SCR, calculated at the reactor inlet, changes in the investigated cases in the range 1.6 ÷ 2.8 to maintain the value of 3 at the beginning of the membrane region.

**Figure 11:**
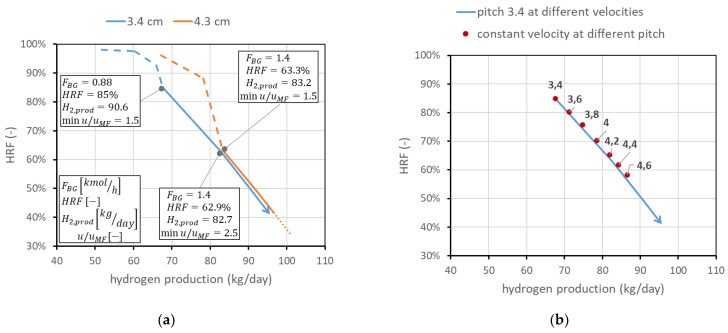
Effect of spacing between the membranes. (**a**) Comparison at different velocities for two different pitches. (**b**) Points at the same velocity (min $u/u_{mf}$ = 1.5) for different pitches (in cm) compared to the line at 3.4 cm pitch and different velocities.

The second analysis concerns membrane spacing. It has been stated that the minimum spacing is bounded to 2 cm due to experimental considerations on CP losses. However, it is still possible to operate at higher spacings. This means, once the geometry and number of membranes are fixed, increasing the reactor diameter. This leads, for a fixed feed flow rate, to a reduction in the gas velocity and then an increase in the residence time, with benefits for the conversion. The line in the performance chart moves towards higher productions and lower HRFs, as shown in Figure 11a for the pitch of two different membranes. The possible spacings, once the operating conditions are fixed, are limited, since the spacing value limits the maximum HRF. A potential interesting range can be from 3.4 to 4.6. To understand how the line moves for different spacings, in Figure 11b, the points representing the operating conditions at minimum $u/u_{mf\;}$= 1.5 for different spacings are plotted in comparison with the line for 3.4 cm pitch at different velocities. The analysis is limited to the solid-line region (i.e., SCR = 3). From each point, it is of course possible to operate at different velocities, producing a line similar to the one obtained for 3.4 cm spacing.

**Figure 12:**
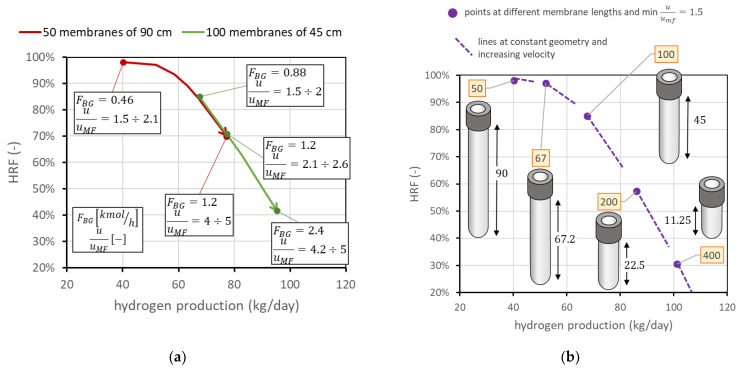
(**a**) Comparison of performance with same total membrane area using 100 membranes of 45 cm length (nominal values) against 50 membranes of 90 cm length. (**b**) Positions of the point with minimum $u/u_{mf}$ = 1.5 for different membrane lengths and numbers, always considering the same total membrane area. Labels indicate the number of membranes.

The third analysis considers the case in which membrane length is not fixed, and then an additional degree of freedom appears in the problem. This is also the case if two membranes can be attached in series to form a longer membrane, which is something still difficult to obtain with ceramic supports, since there could be the presence of leakage in the seals, but that is potentially very interesting with metallic-supported membranes. In this case, is important to understand the difference in having the same total membrane area considering, for example, half the number of membranes with doubled length. For the nominal case, this means comparing the FBMR with another reactor containing 50 membranes of 90 cm length, with the same spacing as that in nominal conditions. Having 50 membranes with a 3.4 cm pitch leads to a reactor diameter of 29 cm, while that in the nominal case is 40 cm. The distance from the distributor plate to the beginning of the membrane region is always fixed at 5 cm. A reactor with longer membranes allows, at the same total membrane area, lower diameters; then, at the same molar flow rate of BG, steam, and air, the velocity is higher than that in the reactor with a bigger diameter. This means that, on the chart, it is also possible to move toward points with higher HRF and lower hydrogen production. From a graphic point of view, shown in Figure 12a, the same membrane area with doubled length leads to the line moving towards the top-left corner, by maintaining the same trend. This discussion can be generalized to different cases at different membrane lengths. Always assuming that the total membrane area is equal to the nominal value (1.98 m^2^), Figure 12b reports the points corresponding to different lengths: 50 membranes of 90 cm, 67 membranes of 67.2 cm, 100 membranes of 45 cm (nominal), 200 membranes of 22.5 cm, or 400 membranes of 11.25 cm. The points in the figure represent the condition at minimum fluidization acceptable ($u/u_{mf}$ = 1.5). From each of them, it is again possible to change the gas velocity, increasing the amount of biogas fed. In Figure 12, lines at different velocities for each geometry are dotted for clarity in representation, but they have the same meaning as the solid lines in the figure on the right side. They represent the possible working points at constant geometry and operating conditions. The fact that each line crosses the other points means that the same point on the chart represents different conditions with different fluidization velocities in different reactors. The longer the membranes, the higher the value of $u/u_{mf}$.

## 4. Reactor Design

In the previous sections, operating and geometric parameters were changed, one at a time, to investigate their impact on the reactor performance. In this section, guidelines on the choice of reactor design are provided based on the previous results.

It is important to state preliminarily that, for the design of the reactor, the HRF has two different upper bounds. The first reason is due to the form of permeation Equation (A6) in Appendix A. Even if all methane available for reforming is converted, and with an infinite membrane area available, it is not possible to separate all the hydrogen produced and then end up with a unitary HRF. This is because pressure in the vacuum side is in any case higher than zero, and then the driving force is zero when hydrogen partial pressure at the membrane surface in the reactor reaches the same value as the permeate-side pressure. Then, higher permeate-side pressures lead to a lower maximum HRF that can be reached with 100% conversion and with an infinite membrane area. This maximum HRF also depends on the retentate pressure, which affects the value of the hydrogen partial pressure and then its molar fraction. At 12 bar and 500 °C, in ATR conditions, the maximum value that can be reached with a complete methane conversion and an infinite membrane area is around 98%. Beyond this, another consideration limits the maximum HRF than can be reached in the reactor: it is in general convenient to recover the heat of the retentate and of the hot permeate to preheat biogas and air and to produce steam for the reactor. To guarantee that permeate and retentate contain enough thermal power, HRF should be limited to an upper limit. The limitation of HRF guarantees that enough hydrogen, methane, and carbon monoxide end up in the retentate, such that their overall LHV can satisfy the heat duty for biogas, air, and steam heating up to the feed temperature. The thermal balance to be guaranteed is reported in Equations (4)–(6). Thermal power available is due to retentate oxidation (LHV) and cooling, together with permeate cooling. The cooling changes from reactor temperature up to the minimum temperature of the feed mixture to be preheated, plus a certain pinch point in the exchanger, assuming $\Delta T_{pp}=20$ °C. On the feed side, biogas, air, and steam should be preheated up to the feed temperature, which is 400 °C. The initial temperature of biogas and air is 140 °C since this is a realistic value of the temperature at their compressor outlet. For steam production, water can be considered already at 120 °C since the heating from ambient temperature to this value can be obtained by recovering the heat released by intercooled compressors of biogas, air, and hydrogen, assuming again $\Delta T_{pp}=20$ °C.

(4) $${\dot{Q}}_{available}\geq {\dot{Q}}_{preheat}$$

(5) $${\dot{Q}}_{available}={\dot{m}}_{ret}\cdot \left[\int_{T_{min,pre\;}+\;\Delta T_{pp}}^{T_{R}}c_{p,ret}\cdot dT+LHV_{ret}\right]+{\dot{m}}_{H_{2},perm}\cdot \int_{T_{min,pre\;}+\;\Delta T_{pp}}^{T_{R}}c_{p,permeate}\cdot dT$$

(6) $${\dot{Q}}_{preheat}={\dot{m}}_{BG}\cdot \int_{T_{out,\;BG\;cmp}}^{T_{feed}}c_{p,BG}\cdot dT+{\dot{m}}_{air}\cdot \int_{T_{out,\;air\;cmp}}^{T_{feed}}c_{p,air}\cdot dT+{\dot{m}}_{H_{2}O}\cdot \left(h_{H_{2}O\;}\left(T_{feed},\;p_{R}\right)-h_{H_{2}O}\left(T_{out,cmp}-\Delta T_{pp},p_{R}\right)\right)$$

The values of maximum HRF in a theoretical calculation (not considering real heat exchangers, but only the total amount of thermal power in retentate and permeate compared to the duty of feed preheating up to 400 °C) depend slightly on reactor pressure and a bit more on reactor temperature, due to variations in retentate LHV. The results of the calculations are reported in Table 4. In the design of the reactor, it should be considered that depending on the heat management and permeate pressure, the HRF has to be set at an appropriate value.

Once the maximum value of HRF is set, the reactor design depends on the specific constraints, mainly due to the membrane production process and hydrogen requirements. As an example, the following analysis will consider a case in which (i) hydrogen production is set to the value of 100 kg_H2_/day (about 46.3 Nm^3^/h); (ii) SCR is optimized from previous analysis on the catalyst performance, and then should be set to the value of 3; (iii) reactor operating pressure is fixed at the value of 12 bar; (iv) reactor operating temperature is 500 °C to guarantee a long stability of the DS membranes; (v) distance from the distributor plate to the beginning of the membranes is set to 5 cm; (vi) permeate-side pressure is fixed at the value of 0.1 bar; (vii) the influence of CP losses is negligible; (viii) the desired fluidization regime is a low-fluidization condition, with minimum $u/u_{mf}$ ratio set to the value of 1.5; (ix) membrane spacing is fixed at the minimum pitch (3.4 cm); and (x) $T_{feed}$ is set to 400 °C. Considering all these constraints, the correct geometry can be identified to reach the maximum HRF starting from considerations coming from the previous section. In the selected operating conditions, as a preliminary calculation, it is possible to set the desired HRF to 92%.

**Figure 13:**
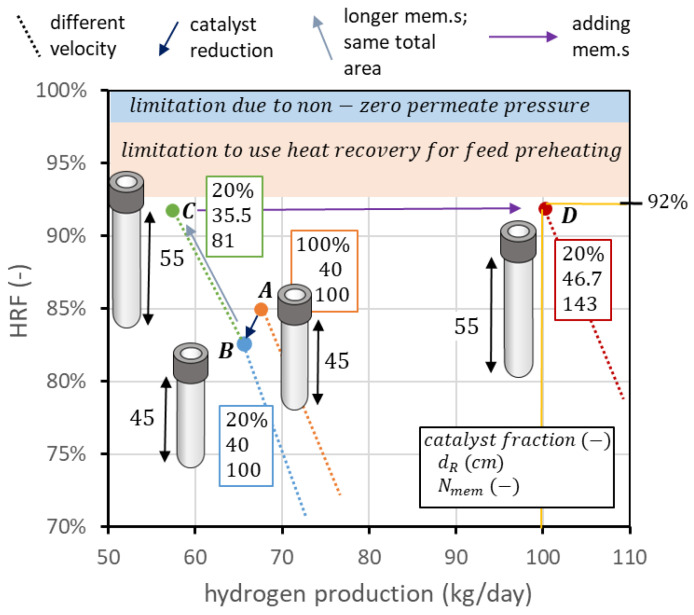
Design procedure to apply the knowledge from reactor analysis to move on the performance chart up to the desired combination of hydrogen production and HRF.

The starting point can be the curve obtained in nominal conditions, considering the higher point of the solid line, which respects the constraints on spacing, $u/u_{mf}$ value, and SCR value. However, this point, which can be seen for example in Figure 4b, has a hydrogen production of 67.6 kg/day and an HRF of 85%. The first design consideration is about the catalyst: in this geometry, about 58 kg of solids with the assumed density and diameter should be filled in the reactor. If all of them are catalytic particles, the associated cost can be very high. Thus, a preliminary sensitivity analysis can be performed on the percentage of catalytic particles over total solids. The results of the analysis, reported in Figure 13a, show that a value of 20% can be selected, since hydrogen production is reduced to 65.7 and HRF to 82.3%, saving 80% of the catalyst cost. To go from this recovery factor to 92%, a possibility is to distribute the same membrane area, but using fewer and longer membranes, as shown in Figure 12. In this procedure, the reactor diameter is reduced as well to maintain the same membrane pitch. If the membrane length is increased by 10 cm, to maintain the same area, the number of membranes changes from 100 to 81. The reactor diameter changes from 40 cm to 35.5 cm. It is then possible to decrease the biogas flow, reaching the point with an HRF of 91.7%, close enough to the target value. The hydrogen production is decreased to 57.4 kg/day. Once the target recovery factor is reached, it is possible to increase hydrogen production with the procedure reported in Figure 10. To produce from 57.4 kg/day to 100 kg/day, the increase in production is 1.74 times. If the membrane area increases by the same factor, maintaining the same membrane length (so adding more membranes and maintaining the same spacing), it is possible to shift the point to the right in the performance chart and then maintain the same recovery factor while increasing hydrogen production. The membrane area changes from 1.98 m^2^ to 3.45 m^2^. With membranes of 55 cm in length and 1.4 cm in diameter, this leads to 143 membranes. The diameter that allows fitting these membranes with a pitch of 3.4 cm is 46.7 cm. The final biogas, steam, and air flow rates are 1.22, 1.25, and 1.32 kmol/h, respectively. The same procedure is illustrated in Figure 13b: starting from the point, at nominal conditions, with minimum $u/u_{mf}$ = 1.5 (point A), catalyst amount is reduced from 100% to 20% of the total solid particles, maintaining the same flow rates, operating conditions, and geometry, ending up at point B. To increase HRF, the same membrane area is distributed in membranes 10 cm longer, going from 100 to 81 membranes and a reactor diameter from 40 to 35.5 cm, to maintain the same spacing. This results in point C. Once HRF is equal to its target value, hydrogen production can be increased up to the target value (100 kg_H2_/day) simply by adding membranes and maintaining the same spacing. This is represented by the arrow going from point C to point D, which is the target value of the design.

It is worth mentioning that the target point identified in the performance chart (100 kg_H2_/day at 92% HRF) has been obtained for a minimum $u/u_{mf}$ of 1.5, but the same point on the chart can be obtained for any fluidization velocity, maintaining the same flow rates of BG, steam, and air, by simply using the same membrane area but with a lower number of longer membranes. Indeed, it is clear from Figure 12a that the curve with longer membranes, at different velocities, passes through all the points, obtained at a lower velocity, of a curve with shorter membranes but the same total membrane area. Then, the same procedure is valid for any fluidization velocity range selected by the designer.

**Figure 14:**
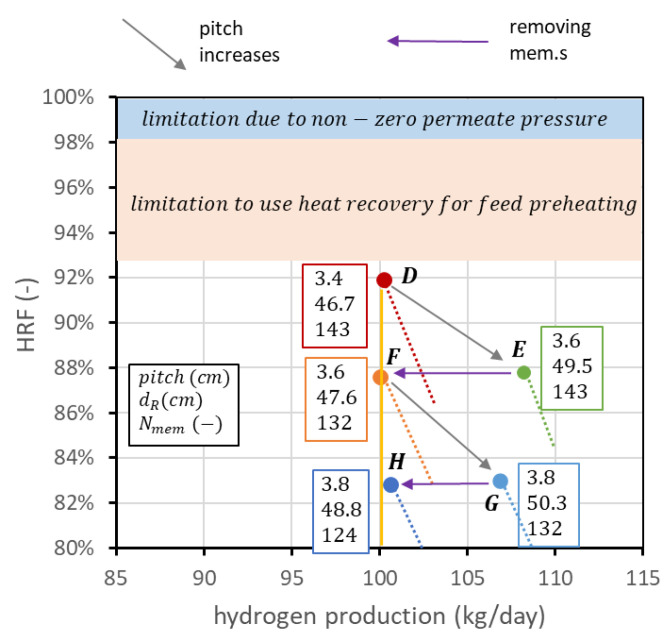
Creation of a set of points (D, F, H) that matches the target production of hydrogen for different combinations of HRF and number of membranes.

The reactor design procedure presented so far is purely technical and depends on the point of view of the reactor only. In industrial practice, the FBMR is inserted in a plant where auxiliaries are necessary for its operation, such as a biogas and air compressor, water pump and components for steam generation, a vacuum pump to produce sub-atmospheric pressure inside the membrane tube, and a hydrogen compressor for final distribution. Performance charts allow an easier understanding of how to operate with the degrees of freedom to obtain a certain set of performance indicators. However, they cannot be used by themselves to compare the layouts at different operating pressures and temperatures since analyses at the system level are necessary to estimate heat recovery and auxiliary consumption. Moreover, even if operating conditions have been set, it is not guaranteed a priori that the point at maximum HRF is the best solution to obtain the desired fixed hydrogen production. This depends in general on the cost of biogas and the cost of the membranes. To explain this concept, it is still possible to refer to point D in Figure 13, following the steps illustrated in Figure 14. Starting from D, it is possible to increase the membrane pitch, keeping all the other parameters constant. This means increasing the reactor diameter while the number of membranes is constant. The point with the same fluidization regime ($\min u/u_{mf}=1.5$) with a pitch of 3.6 cm is point E. In this case, membrane area is not changed, hydrogen production is increased, and HRF is decreased. From E, it is possible to reduce the number of membranes to maintain the new HRF, coming back to the set production. The point with the new pitch and 100 kg_H2_/day is point F. Going from D to F, HRF has been reduced from 92 to 88%; thus, biogas cost is increased, but the number of membranes is decreased from 143 to 132. With exactly the same procedure, it is possible to go from point F to point G and then to point H. In this way, a set of points (D, F, H) with the target production of hydrogen but different combinations of HRF and number of membranes can be created. It is of course possible to repeat the procedure and add other points. In general, even if the final decision should be evaluated from an analysis at the system level, due to the high biogas cost, it is better to design the reactor with the highest HRF possible. The analysis can be interesting in case of a high membrane cost and low-cost biogas availability.

## 5. Outlooks

Modelling activity for FBMR can be further refined to investigate some effects that have been neglected in this work. The most important is the estimation of the CP losses, for which only a potential range has been provided. Another effect can be the consideration of how the membranes perform depending on their geometrical configuration. In this work, it has been assumed that all the membranes perform in the same way, while in reality, performance can be different if the membrane is at the center or at the edge of the pattern. Experimental results so far have been obtained only in lab-scale reactors, so the effect at the investigated scale is still unknown. The MACBETH project and the FBMR prototype will allow answering these questions.

## 6. Conclusions

The fluidized bed membrane reactor is a promising technology for the small-scale production of green hydrogen from biogas. This technology is close to industrial maturity, and more information about its behavior is required to successfully predict its performance and to develop the scaling up.

In this article, some guidelines on FBMR design and operation have been provided through investigations on the influence of the most relevant parameters. Results are shown in performance charts, which allow the possible operating conditions and the influence of design and operating parameters to be understood. The reactor temperature is always beneficial from a thermodynamic point of view, while pressure has a trade-off between efficiency and hydrogen production. So, for example, to work at partial load, it is convenient to reduce the reactor pressure since HRF increases. Other variables have a saturation effect that should be accounted for to avoid ineffective costs, such as permeate-side pressure and the amount of catalyst. The latter also influences the shape of the curve in the performance chart, so a deactivation behavior can be also detected and discerned from other phenomena. It is important to remark that CP losses can have a big influence on performance.

## Figures and Tables

**Table 1:** BG and air compositions.

Species	Biogas (%mol)	Air (%mol)
$CH_{4}$	58.1	0
$CO_{2}$	33.9	0
$N_{2}$	3.8	79
$O_{2}$	1.1	21
$H_{2}O$	3.1	0

**Table 2:** Double-skin membranes parameters for permeation equation.

Parameter	Value	Units
$P_{H_{2}}^{0}$	$5.87\cdot 10^{-10}$	$\mathrm{mol}/\left(s\cdot m\cdot {\mathrm{bar}}^{0.5}\right)$
$E_{a,perm}$	7.81	kJ/mol
n	0.749	-
$t_{SL}$	2.5	μm
$k_{m}f$	79.2	m/h

**Table 3:** Catalyst parameters for reaction rate equations.

Parameter	Value	Units
$k_{SMR}^{0}$	$3.492\cdot 10^{5}$	$\mathrm{kmol}/\left(h\cdot \mathrm{kg}\cdot {\mathrm{bar}}^{0.404}\right)$
$E_{a,SMR}$	83.6	kJ/mol
$k_{WGS}^{0}$	$6.192\cdot 10^{3}$	kmol/(h·kg·bar)
$E_{a,WGS}$	54.5	kJ/mol
$\rho_{p}$	2095	kg/m^3^
$d_{p}$	204	μm

**Table 4:** Maximum HRF for different reactor temperatures and pressures to guarantee enough thermal power for feed preheating.

$p_{R}\;\left(\mathrm{bar})/T_{R}\;({}^{\circ}C\right)$	475	500	525
9	89.7	91.3	93.6
12	90.3	92.0	93.7
15	90.7	92.4	94.1

## Nomenclature

Where x appears in the unit column, it means that the unit depends on the specific reaction or component.

Parameter	Description	Unit
A	Cross-section area	m^2^
Ar	Archimedes number	-
b	Membrane pitch	m
c	Molar concentration	kmol/m^3^
$c_{p}$	Specific heat at constant pressure	kJ/(kmol∙K)
d	Diameter	m
D	Diffusivity	m^2^/s
$E_{a}$	Activation energy	kJ/mol
$F_{H_{2}}$	Total moles of hydrogen permeated at each axial position z	kmol/(h∙m)
*g*	Gravitational acceleration	m/s^2^
h	Molar enthalpy	kJ/kmol
HRF	Hydrogen recovery factor	-
$J_{H_{2}}$	Hydrogen flux through the membrane	kmol/(h∙m^2^)
$k_{m}f$	Mass transfer coefficient in film layer model	m/h
$k^{0}$	Pre-exponential coefficient in chemical reaction	kmol/(h∙kg∙bar^x^)
K	Exchange coefficient between FBMR phases	1/h
$K_{eq}$	Equilibrium constant of chemical reaction	bar^x^
LHV	Lower heating value	MJ/kg
$\dot{m}$	Mass flow rate	kg/s
n	Exponent in hydrogen flux expression	-
$\dot{n}$	molar flow rate	kmol/h
$N_{mem}$	Number of membranes	-
NR	Number of chemical reactions considered	-
p	Pressure	bar_a_
$p_{i}$	Partial pressure of component *i*	bar
$\;P_{H_{2}}^{0}$	Pre-exponential factor of membrane permeability	mol/(s∙m∙bar^0.5^)
$r_{R.j}$	Reaction rate of reaction *j*	kmol/(h∙kg_cat_)
R	Universal gas constant	kJ/(mol∙K)
SCR	Ratio between moles of steam and moles of methane fed	-
t	Thickness	m
T	Temperature	°C
u	Velocity	m/s
${\dot{V}}_{total}$	Total volumetric flow rate of gas through the reactor	m^3^/h
$x_{H_{2}}$	Molar fraction of hydrogen	-
z	Axial coordinate along the reactor	m
Greek letter		
α	Parameter for wake fraction estimation	-
δ	Fraction of phase (wake or emulsion or bubble)	-
Δ	Difference	x
ε	Void fraction	-
$\nu_{j,i}$	Stoichiometric coefficient of species i in reaction j	-
μ	Viscosity	Pa∙s
ρ	Density	kg/m^3^
subscripts		
0	Reference size or variable at position z=0 in the reactor	
b	Parameter in bubble phase	
e	Parameter in emulsion phase	
feed	Parameter valid for the feed stream	
g	Parameter averaged in gas phase flowing into the reactor	
i	Parameter valid for chemical component (or species) i	
in	Parameter at the reactor/system inlet	
*j*	Parameter related to chemical reaction j	
mem	Parameter related to the membranes	
mem surface	Parameter calculated at the membrane surface	
mf	In minimum fluidization conditions	
ox	Oxidated	
p	Parameter of a single catalyst particle	
pp	Pinch-point in heat exchangers	
perm	Related to the permeation process	
permeate	Parameter about the permeate stream	
R	Parameter value for the reactor	
*ret*	Parameter for the retentate stream	
SL	Selective layer of the membrane	
SMR	Steam methane reforming reaction	
w	Parameter in wake phase	
WGS	Water gas shift reaction	

## Acronyms

1D	Mono-dimensional
ACM	Aspen Custom Modeler
ATR	Autothermal reforming
BG	Biogas
CCUS	Carbon capture, utilization, and storage
CP	Concentration polarization
EU	European Union
FBMR	Fluidized bed membrane reactor
HRF	Hydrogen recovery factor
KPI	Key performance indicator
LHV	Lower heating value
MACBETH	Membranes And Catalysts Beyond Economic and Technological Hurdles
MR	Membrane reactor
SCR	Steam–carbon ratio

## Appendix A. FBMR Model Equations

The FBMR model is based on a steady-state material balance for all the chemical components involved. The model is 1D along the reactor length (coordinate *z*).

(A1) $$\frac{\partial {\dot{n}}_{i}\left(z\right)}{\partial z}=A_{R}\left(z\right)\cdot \rho_{p}\cdot \left(1-\varepsilon_{mf}\left(z\right)\right)\cdot \delta_{e/w}\cdot \overset{NR}{\underset{j}{\sum }}r_{R.j}\left(z\right)\cdot \nu_{j,i}\pm A_{R}\left(z\right)\cdot \delta_{b}\left(z\right)\cdot K_{be}\left(z\right)\cdot \left(c_{i,b}\left(z\right)-c_{i,e}\left(z\right)\right)-F_{H_{2},perm}\left(z\right)\;$$

Variation in the molar flow of chemical component *i* depends on generation and/or consumption in chemical reactions ($\delta_{e}$ if the balance is in the emulsion phase, $d_{w}$ if it is in the bubble phase), exchange between bubble and emulsion phase (plus sign if the balance is done in the bubble phase, minus if is done in the emulsion phase), and moles permeated (if the component is hydrogen). Reaction rates of reactions R.1 and R.2 are as follows:

(A2) $$r_{R.1}\left(z\right)=\frac{k_{\mathrm{SMR}}^{0}\cdot \exp\left(\frac{-E_{a,\mathrm{SMR}}}{R\cdot T_{R}}\right)\cdot \left(p_{{\mathrm{CH}}_{4}}\left(z\right)\cdot p_{H_{2}O}\left(z\right)-\frac{p_{H_{2}}^{3}\left(z\right)\cdot p_{\mathrm{CO}}\left(z\right)}{K_{\mathrm{eq},\mathrm{SMR}}}\right)}{p_{H_{2}O}^{1.596}\left(z\right)}$$

(A3) $$r_{R.2}\left(z\right)=\frac{k_{WGS}^{0}\cdot \exp\left(\frac{-E_{a,WGS}}{R\cdot T_{R}}\right)\cdot \left(p_{CO}\left(z\right)\cdot p_{H_{2}O}\left(z\right)-\frac{p_{H_{2}}\left(z\right)\cdot p_{CO_{2}}\left(z\right)}{K_{eq,WGS}}\right)}{p_{H_{2}O}\left(z\right)}$$

It is assumed that reaction R.3 happens instantly at the reactor inlet since combustion is generally very fast. Values of the parameters used in the kinetic model can be found in [21].

The total amount of hydrogen separated, and then pure hydrogen produced from the plant, can be calculated from the amount permeated at each position by integrating the permeation along the membrane starting and end positions:

(A4) $${\dot{n}}_{H_{2,perm}}=\int_{start\;mem}^{end\;mem}F_{H_{2},perm}\left(z\right)\cdot dz$$

The amount of hydrogen permeated at position z can be calculated from the hydrogen flux:

(A5) $$F_{H_{2},perm}\left(z\right)=N_{mem}\cdot \pi \cdot d_{mem}\cdot J_{H_{2},perm}\left(z\right)$$

Hydrogen flux is obtained from Richardson’s equation:

(A6) $$J_{H_{2},perm}\left(z\right)=\frac{P_{H_{2}}^{0}\cdot \exp\left(\frac{-E_{a,perm}}{R\cdot T_{R}}\right)}{t_{SL}}\cdot \left(p_{H_{2},R}^{n}\left(z\right)-p_{H_{2},permeate}^{n}\right)$$

Parameters for the permeation equation are reported in Table 2.

If concentration polarization losses are taken into account, the hydrogen molar fraction at the membrane surface is calculated using the film layer model:

(A7) $$J_{H_{2},perm}\left(z\right)=k_{m}f\cdot c_{g}\cdot \ln\left(\frac{1-x_{H_{2,\;\;mem\;surface}}}{1-x_{H_{2,R}}}\right)$$

An overall energy balance is also included to guarantee the autothermal behavior of the reactor:

(A8) $${\dot{n}}_{feed}\cdot h_{feed}={\dot{n}}_{ret}\cdot h_{ret}+{\dot{n}}_{H_{2,perm}}\cdot h_{permeate}$$

The model is completed with the fluid dynamic description of the fluidized bed. Relations are mainly taken from [4].

(A9) $$Ar\left(z\right)=\frac{d_{p}^{3}\cdot \rho_{g}\left(z\right)\cdot \left(\rho_{p}-\rho_{g}\left(z\right)\right)\cdot g}{\mu_{g}^{2}\left(z\right)}$$

(A10) $$\varepsilon_{mf}\left(z\right)=0.586\cdot Ar^{-0.029}\left(z\right)\cdot {\left(\frac{\rho_{g}\left(z\right)}{\rho_{p}}\right)}^{0.021}$$

(A11) $$u_{mf}\left(z\right)=\left(\frac{\mu_{g}\left(z\right)}{d_{p}\cdot \rho_{g}\left(z\right)}\right)\cdot \left(\sqrt{{\left(27.2\right)}^{2}+0.0408\cdot Ar\left(z\right)}-27.2\right)$$

$$u\left(z\right)=\frac{{\dot{V}}_{total}\left(z\right)}{A_{R}\left(z\right)}\;$$

(A12) $$d_{b,0}=0.376\cdot {\left(u\left(0\right)-u_{mf}\left(0\right)\right)}^{2}$$

(A13) $$d_{b,max}\left(z\right)=\min\left(d_{R}\left(z\right);0.65\cdot {\left(\frac{\pi }{4}\cdot d_{R}^{2}\left(z\right)\cdot \left(u\left(z\right)-u_{mf}\left(z\right)\right)\right)}^{0.4}\right)$$

(A14) $$d_{b}\left(z\right)=d_{b,max}\left(z\right)-\left(d_{b,max}\left(z\right)-d_{b,0}\right)\cdot \exp\left(-\frac{0.3\cdot z}{d_{R}\left(z\right)}\right)$$

(A15) $$u_{b}\left(z\right)=u\left(z\right)-u_{mf}\left(z\right)+0.711\cdot {\left(g\cdot d_{b}\left(z\right)\right)}^{0.5}$$

(A16) $$\delta_{b}\left(z\right)=\frac{u\left(z\right)-u_{mf}\left(z\right)}{u_{b}\left(z\right)}$$

(A17) $$\delta_{w}\left(z\right)=\alpha \left(z\right)\cdot \delta_{b}\left(z\right)$$

(A18) $$\alpha \left(z\right)=1-\exp\left(-4.92\cdot d_{b}\left(z\right)\right)$$

(A19) $$\delta_{e}=1-\left(\delta_{b}+\delta_{w}\right)$$

(A20) $$K_{b}\left(z\right)=4.5\cdot \left(\frac{u_{mf}\left(z\right)}{d_{b}\left(z\right)}\right)+5.85\cdot \left(\frac{D_{g}^{0.5}\left(z\right)\cdot g^{0.25}}{d_{b}^{1.25}\left(z\right)}\right)\cdot 3600$$

(A21) $$K_{e}\left(z\right)=6.77\cdot {\left(\frac{D_{g}\left(z\right)\cdot u_{b}\left(z\right)\cdot \varepsilon_{mf}\left(z\right)}{d_{b}^{3}}\right)}^{0.5}\cdot 3600$$

(A22) $$K_{be}\left(z\right)={\left(\frac{1}{K_{b}\left(z\right)}+\frac{1}{K_{e}\left(z\right)}\right)}^{-1}$$

For reactor geometry, the maximum number of membranes fitting into a reactor of diameter $d_{R}$ with a pitch b is a polynomial relation taken from [26] and results in the following:

(A23) $$N_{mem}=0.7854\cdot {\left(\frac{d_{R}}{b}\right)}^{2}-0.2349\cdot \left(\frac{d_{R}}{b}\right)-2.1429$$

## References

[1] Gallucci F., Tanaka D.P., Medrano J.A., Sole J.V. (2020). Membrane Reactors Using Metallic Membranes. Current Trends and Future Developments on (Bio-) Membranes.

[2] MACBETH » Macbeth. https://www.macbeth-project.eu/.

[3] Gallucci F., Fernandez E., Corengia P., van Sint Annaland M. (2013). Recent advances on membranes and membrane reactors for hydrogen production. Chem. Eng. Sci..

[4] Kunii D., Levenspiel O. (1991). Fluidization Engineering.

[5] Di Marcoberardino G., Binotti M., Manzolini G., Viviente J.L., Arratibel A., Roses L., Gallucci F. (2017). Achievements of European projects on membrane reactor for hydrogen production. J. Clean. Prod..

[6] BIONICO Project. http://www.bionicoproject.eu/.

[7] International Energy Agency (2022). Global Hydrogen Review 2022. www.iea.org/t&c/.

[8] International Energy Agency (2019). The Future of Hydrogen: Seizing Today’s Opportunities. https://www.iea.org/reports/the-future-of-hydrogen.

[9] Gas for Climate (2020). Market State and Trends in Renewable and Low-Carbon Gases in Europe. https://www.europeanbiogas.eu/wp-content/uploads/2020/12/GfC_MSTReport_2020_final.pdf.

[10] Marra L., Wolbers P.F., Gallucci F., van Sint Annaland M. (2014). Development of a RhZrO_2_ catalyst for low temperature autothermal reforming of methane in membrane reactors. Catal. Today.

[11] Brencio C., Fontein F.W.A., Medrano J.A., Di Felice L., Arratibel A., Gallucci F. (2022). Pd-based membranes performance under hydrocarbon exposure for propane dehydrogenation processes: Experimental and modeling. Int. J. Hydrogen Energy.

[12] Medrano J.A., Tasdemir M., Gallucci F., van Sint Annaland M. (2017). On the internal solids circulation rates in freely-bubbling gas-solid fluidized beds. Chem. Eng. Sci..

[13] de Nooijer N., Gallucci F., Pellizzari E., Melendez J., Tanaka D.A.P., Manzolini G., van Sint Annaland M. (2018). On concentration polarisation in a fluidized bed membrane reactor for biogas steam reforming: Modelling and experimental validation. Chem. Eng. J..

[14] Helmi A., Voncken R.J.W., Raijmakers A.J., Roghair I., Gallucci F., van Sint Annaland M. (2018). On concentration polarization in fluidized bed membrane reactors. Chem. Eng. J..

[15] Gallucci F., van Sint Annaland M., Kuipers J.A.M. (2008). Autothermal Reforming of Methane with Integrated CO_2_ Capture in a Novel Fluidized Bed Membrane Reactor. Part 2 Comparison of Reactor Configurations Number of CSTRs in the bubble phase N e Number of CSTRs in the emulsion phase. Top. Catal..

[16] Deshmukh S.A.R.K., Laverman J.A., Cents A.H.G., van Sint Annaland M., Kuipers J.A.M. (2005). Development of a Membrane-Assisted Fluidized Bed Reactor. 1. Gas Phase Back-Mixing and Bubble-to-Emulsion Phase Mass Transfer Using Tracer Injection and Ultrasound Experiments. Ind. Eng. Chem. Res..

[17] Brencio C., Di Felice L., Gallucci F. (2022). Fluidized Bed Membrane Reactor for the Direct Dehydrogenation of Propane: Proof of Concept. Membranes.

[18] Foresti S., Di Marcoberardino G., Manzolini G., De Nooijer N., Gallucci F., van Sint Annaland M. (2018). A comprehensive model of a fluidized bed membrane reactor for small-scale hydrogen production. Chem. Eng. Process.-Process Intensif..

[19] Di Marcoberardino G., Knijff J., Binotti M., Gallucci F., Manzolini G. (2019). Techno-Economic Assessment in a Fluidized Bed Membrane Reactor for Small-Scale H_2_ Production: Effect of Membrane Support Thickness. Membranes.

[20] Di Marcoberardino G., Gallucci F., Manzolini G., van Sint Annaland M. (2016). Definition of validated membrane reactor model for 5 kW power output CHP system for different natural gas compositions. Int. J. Hydrogen Energy.

[21] Ongis M., Di Marcoberardino G., Manzolini G., Gallucci F., Binotti M. (2023). Membrane reactors for green hydrogen production from biogas and biomethane: A techno-economic assessment. Int. J. Hydrogen Energy.

[22] Spallina V., Pandolfo D., Battistella A., Romano M.C., Annaland M.V.S., Gallucci F. (2016). Techno-economic assessment of membrane assisted fluidized bed reactors for pure H_2_ production with CO_2_ capture. Energy Convers. Manag..

[23] Di Marcoberardino G., Foresti S., Binotti M., Manzolini G. (2018). Potentiality of a biogas membrane reformer for decentralized hydrogen production. Chem. Eng. Process.-Process Intensif..

[24] Arratibel A., Tanaka A.P., Laso I., van Sint Annaland M., Gallucci F. (2018). Development of Pd-based double-skinned membranes for hydrogen production in fluidized bed membrane reactors. J. Memb. Sci..

[25] Voncken R.J.W., Roghair I., van Sint Annaland M. (2019). A numerical study on concentration polarization in 3D cylindrical fluidized beds with vertically immersed membranes. Chem. Eng. Sci..

[26] Smaller Circles within a Larger Circle-Calculator. https://www.engineeringtoolbox.com/smaller-circles-in-larger-circle-d_1849.html.

